# Using the Sonification for Hardly Detectable Details in Medical Images

**DOI:** 10.1038/s41598-019-54080-7

**Published:** 2019-11-27

**Authors:** Veturia Chiroiu, Ligia Munteanu, Rodica Ioan, Ciprian Dragne, Luciana Majercsik

**Affiliations:** 1 Institute of Solid Mechanics of Romanian Academy, Department of Deformable Media and Ultrasonics, Ctin Mille 15, Bucharest, 010141 Romania; 2 University Spiru Haret, Bucharest, Department of Mathematics, 13 Str. Ion Ghica, Bucharest, 030045 Romania; 3 University Transilvania of Brasov, B-dul Eroilor nr. 29, Brașov, 500036 Romania

**Keywords:** Biomedical engineering, Computer science

## Abstract

The inverse sonification problem is investigated in this article in order to detect hardly capturing details in a medical image. The direct problem consists in converting the image data into sound signals by a transformation which involves three steps - data, acoustics parameters and sound representations. The inverse problem is reversing back the sound signals into image data. By using the known sonification operator, the inverse approach does not bring any gain in the sonified medical imaging. The replication of the image already known does not help the diagnosis and surgical operation. In order to bring gains in the medical imaging, a new sonification operator is advanced in this paper, by using the Burgers equation of sound propagation. The sonified medical imaging is useful in interpreting the medical imaging that, however powerful they may be, are never good enough to aid tumour surgery. The inverse approach is exercised on several medical images used to surgical operations.

## Introduction

A significant effort has been devoted in recent years to improve the quality of medical images used to surgery^1^. The Roentgen’s discovery of X-rays (1895) was followed by the computed tomography, magnetic resonance imaging, nuclear imaging, and ultrasound-positioned medical imaging used for diagnoses and surgery.

To our knowledge, we are the first to apply the sonification theory to uncover hidden details in medical images, such as vessels, organs or tumors that cannot be directly seen with the eye. The beginnings of the sonification theory are dated in 1952 when Pollack evaluates auditory displays as a visualization tool by using the information theory^2,3^. The International Community for Auditory Display Conference organized by Kramer in 1992 has generated great interest for this multi-disciplinary theory, from the science and technology to the arts^4,5^. Licht pursues the history of the sound art in 2007 by highlighting ancient art such as Sonic Youth and contemporary art that led to challenging applications, including the works of Christian Marclay, LaMonte Young, Janet Cardiff, Rodney Graham and Laurie Anderson among many others^6^.

The nano-guitar built at Cornell University from crystalline silicon no larger than a single human blood cell, invites bacteria inside a pacient to sing so that it can be easily detectable by a stethoscope^7^. The quantum whistle produces a nano-scale sound capable of detecting oscillations in the superfluid gases predicted by quantum theory^8^.

The sonification theory allows new perspectives in the diagnosis of diseases, such as the Alzheimers’s dementia^9^ and therapies in body movements, such as walking, twisting, lifting the arms or moving the legs^10^. The inverse problem of sonification, that is, the reversal of sound samples in new images is less studied so far, to the best of our knowledge. This is due to the fact that the known sonification operator does not bring any improvement of the medical image, because the theory behind it is the linear theory of sound motion. This article introduces a new sonification operator based on the nonlinear Burgers theory of the sound motion. The new operator has proved its ability to solve the inverse problem of sonification and to obtain essential gains in improving the medical image.

The paper is organized as follows: Section 2 is devoted to description of the direct problem of sonification. A description of the new sonification operator based on the Burgers equation of sound propagation is presented in Section 3. The methodology is described in Section 4, the applications in Section 5, while Section 6 contains the conclusions.

## Direct Problem of Sonification

The direct problem of sonification, as known in the literature^11–14^, is based on a sonification operator *S*^0^ to transform the image point data *D* into sound signals *Y*^0^ as *S*^0^: *D* → *Y*^0^, *S*^0^: *x*(*t*) → *y*^0^(*t*^0^, *x*(*t*), *p*^0^), where *x*(*t*) is a 1D string of point data, *t* is the data time, *t*^0^ is the sonification time, and *p*^0^ ⊆ *P*^0^ is the set of sonification parameters.

The parameters *P*^0^ = {*k*^0^, Δ^0^, ${f}_{ref}^{0}$, α^0^, β^0^, ϕ^0^, ε^0^, *g*^0^, γ^0^, *H*^0^} include *k*^0^, the factor of time compressor on the interval *T*^0^ = *T*/*k*^0^, Δ^0^ ≥ 0, the factor of dilation, ${f}_{ref}^{0}$, the reference frequency, α^0^, β^0^ ≥ 0, the pitch scaling factors, ϕ^0^ ≥ 1, the power distortion factor, ε^0^ ≥ 0, the amplitude threshold, *g*^0^, the gain function, γ^0^, the decay parameter and *H*^0^, the timbral control function.

The variables of the data domain are *t*, *t*_*i*_, *T*. The signal *x*(*t*) can be divided into segments of different length that do not overlap, being expressed as a sequence *x*(*n*) of *N* = *T* × *f*_*s*_ samples at the rate *f*_*s*_ of *T* seconds duration. The time points *t*_*i*_ split in time the segments *x*_*i*_(*t*). A possible division in *M* segments of *x*_*i*_(*t*) is

1 $${x}_{i}(t)=\{\begin{array}{cc}x(t+{t}_{i-1}) & 0\le t\le ({t}_{i}-{t}_{i-1}),\\ 0 & {\rm{else}},\end{array}$$

for *t*_0_ = 0 and *t*_*M*_ = *T*. The duration of each segment is *T*_*i*_ = *t*_*i*_ − *t*_*i*−1_.

Each segment *x*_*i*_(*t*) is sonified as a single event ${y}_{i}^{0}({t}^{0})$ longer or shorter than *T*_*i*_

2 $${y}^{0}({t}^{0})=\mathop{\sum }\limits_{i=1}^{M}{y}_{i}^{0}({t}^{0}-{t}_{i-1}^{0}),\,\,\,{t}_{i-1}^{0}=\frac{{t}_{i-1}}{{k}^{0}}.$$

The general form for the sonified signal *y*^0^(*t*^0^) is

3 $${y}_{i}^{0}({t}^{0})=|{x}_{i}({\Delta }^{0}{t}^{0})|\,\sin \,(2{\rm{\pi }}\,{\int }_{0}^{{t}^{0}}{f}_{ref}{2}^{({x}_{trend}({t}_{i-1})+{x}_{i}({\Delta }^{0}{t}^{0^{\prime} }))}\,d{t}^{0^{\prime} }),$$

where *x*_*i*_(Δ^0^*t*^0′^) is the mean free segment, and *x*_*trend*_(*t*_*i*−1_) is the trend signal at the starting point for pitch modulation. Parameter Δ^0^ gives the length of the event ${T}_{i}^{0}$. If Δ^0^ = *k*^0^ the adjacent events do not overlap but they can overlap for Δ^0^ ≤ *k*^0^.

To introduce control of timbre, the operator *H*^0^ acts as the sine function, so

4 $${y}_{i}^{0}({t}^{0})={a}_{i}({t}^{0}){H}^{0} < \sin \,(2{\rm{\pi }}\,{\int }_{0}^{{t}^{0}}{f}_{ref}{2}^{{b}_{i}({t}^{0^{\prime} }))}\,d{t}^{0^{\prime} }) > ,\,{b}_{i}({t}^{0^{\prime} })=({{\rm{\alpha }}}^{0}{x}_{trend}({t}_{i-1})+{{\rm{\beta }}}^{0}{x}_{i}(\Delta {t}^{0^{\prime} })),$$

where *a*_*i*_(*t*^0^) is the modulation amplitude, *f*_*ref*_ is the base frequency for the pitch range of sonification, and *b*_*i*_(*t*^0^) is a pitch modulator. The amplitude modulator is defined as

5 $${a}_{i}({t}^{0})={|{x}_{i}({\Delta }^{0}{t}^{0}|}^{{\phi }^{0}},\,{\phi }^{0}\ge 1$$

where φ^0^ is the amplitude modulator. A half-wave rectification is included for exceeding a threshold ε^0^ around the mean of the amplitude, a half-wave rectification is included

6 $${a}_{i}({t}^{0})=g(|{x}_{i}({\Delta }^{0}{t}^{0}|,{{\rm{\varepsilon }}}^{0}),\,g(x,{{\rm{\varepsilon }}}^{0})=\{\begin{array}{cc}x-{{\rm{\varepsilon }}}^{0} & x\ge {{\rm{\varepsilon }}}^{0},\\ 0 & {\rm{else}}.\end{array}$$

## New Sonification Operator

We are looking for a new operator to replace (3) based on the Burgers equation of sound propagation. A digital image *B* is seen as a collection of *N* pixels. We suppose that *B* is subjected to external vibration force *f*(*t*) expressed as a sum of harmonic force *F*_*p*_(*t*), and the generation sound force *F*_*s*_(*t*). The force *F*_*s*_(*t*) has the role to build the sonification operator. The response of *B* to *f*(*t*) is a new configuration *b* of all points *P* ∈ *B* at the time *t*. The vibration of *B* is described by Burgers equation^15^

7 $$\frac{\partial v}{\partial x}-\frac{{\rm{\beta }}}{{c}_{0}^{2}}v\frac{\partial v}{\partial {\rm{\tau }}}-\frac{b}{2{{\rm{\rho }}}_{0}{c}_{0}^{3}}v\frac{{\partial }^{2}v}{\partial {{\rm{\tau }}}^{2}}=0,$$

where *x* = (*x*_1_, *x*_2_, *x*_3_) is the position vector, *v* = (*v*_1_, *v*_2_, *v*_3_) is the acoustic velocity vector, τ = *t* − *x*/*c*_0_ is the retarded time, *t* is time, *c*_0_ is the velocity of sound motion in the linear approximation, *b* = (*b*_1_, *b*_2_, *b*_3_) are the dissipation coefficients, ρ_0_ is density of medium, β = (β_1_, β_2_, β_3_) is nonlinearity coefficients. Details on the pulse propagation in nonlinear 1D media can be found in^16–19^.

Equation (7) admits the cnoidal solutions^20^. These solutions are localized waves that conserve their properties even after interaction among them, and then act somewhat like particles. This equation and other equations of the same kind (Schrödinger, Korteweg–de Vries equations etc.) have an infinite number of local conserved quantities, an infinite number of exact solutions expressed in terms of the Jacobi elliptic functions (cnoidal solutions) or the hyperbolic functions (solitons), and the simple formulae for nonlinear superposition of explicit solutions.

Given a known force *F*_*p*_(*t*), we determine *F*_*s*_(*t*) such that the acoustic power *W* radiated from *B* to be minimum. The *W* is written as

8 $$W=\frac{A}{2}{v}^{T}p,$$

where *v* is the velocity verifying (7) and *p* the acoustic pressure vector, *A* is the area of the image, and the subscript *T* represents the Hermitian transpose^21^.

The solutions *v*_*i*_, *i* = 1, 2, 3 of (7) are expressed as

9 $${v}_{i}=\mathop{\sum }\limits_{j=1}^{l}{a}_{j}\,{{\rm{cn}}}^{j}({m}_{i},{{\rm{\eta }}}_{i})+\frac{\mathop{\sum }\limits_{j=1}^{l}{b}_{j}\,{{\rm{cn}}}^{j}({m}_{i},{{\rm{\eta }}}_{i})}{1+\mathop{\sum }\limits_{j=1}^{l}{c}_{j}\,{{\rm{cn}}}^{j}({m}_{i},{{\rm{\eta }}}_{i})},\,i=1,2,3,$$

where ${{\rm{\eta }}}_{i}={k}_{1i}{x}_{1}+{k}_{2i}{x}_{2}+{k}_{3i}{x}_{3}-{{\rm{\omega }}}_{i}t+{\tilde{{\rm{\phi }}}}_{i}$, *l* is a finite number of degree of freedom of the cnoidal functions, 0 ≤ *m* ≤ 1 is the moduli of the Jacobean elliptic function, ω is frequency and $\tilde{{\rm{\phi }}}$ the phase, *k*_1_, *k*_2_, *k*_3_ are components of the wave vector^20^. In the following, we stop to *l* = 2, and we will see that there are no sensible improvements in solutions for *l* > 2 The function *F*_*s*_(*t*) is de*t*ermined from

10 $$\frac{\partial W}{\partial {F}_{s}}=0.$$

The unknown parameters ${P}_{j}=\{{m}_{j},{{\rm{\omega }}}_{j},{k}_{1j},{k}_{2j},{k}_{3j},{\tilde{{\rm{\phi }}}}_{j},{a}_{1},{b}_{1},{c}_{1},{a}_{2},{b}_{2},{c}_{2}\}$, *j* = 1, 2, 3, are find by a genetic algorithm which minimizes the objective function ϒ(*P*_*j*_) written with respect to residuals of (7) and (10)

11 $$\varUpsilon ({P}_{j})={3}^{-1}\mathop{\sum }\limits_{j=1}^{3}{{\rm{\delta }}}_{1j}^{2}+{{\rm{\delta }}}_{2}^{2},$$

with

12 $${{\rm{\delta }}}_{1j}=\frac{\partial {v}_{j}}{\partial {x}_{j}}-\frac{{{\rm{\beta }}}_{j}}{{c}_{0}^{2}}{v}_{j}\frac{\partial {v}_{j}}{\partial {\rm{\tau }}}-\frac{{b}_{j}}{2{{\rm{\rho }}}_{0}{c}_{0}^{3}}{v}_{j}\frac{{\partial }^{2}{v}_{j}}{\partial {{\rm{\tau }}}^{2}},\,{{\rm{\delta }}}_{2}=\frac{\partial W}{\partial {F}_{s}}.$$

The genetic algorithm is running until it is reached a non-trivial minimizer, which will be a point at which (11) admits a global minimum.

The quality of results depends on the values of ϒ. The required precision is taken to be six places after the decimal point. The genetic parameters are assumed to be as follow: number of populations 200, ratio of reproduction 1.0, number of multi-point crossover 1, probability of mutation 0.5, and maximum number of generations 500.

Once determined the function *F*_*s*_(*t*), the sonification operator *S*(*D*, *t*) is written as

13 $$S(D,t)={F}_{s}(t)+\frac{{F}_{s}(t)}{1+{F}_{s}(t)}=\tilde{D}(t),$$

where *D* = {*d*_1_, *d*_2_, …, *d*_*N*_}, *d*_*i*_ ∈ *R*^*N*^ is the point data domain of the original image. The data matrix *D* is obtained from *B* by applying the converter *C*_1_ as *C*_1_(*B*) = *D*. The converter *C*_1_ is defined in the next Section. Data *D* is arranged as a matrix with arbitrarily number of elements.

In (13), $\tilde{D}=\{{\tilde{d}}_{1},{\tilde{d}}_{2},\ldots ,{\tilde{d}}_{N}\},{\tilde{d}}_{i}\in {R}^{N}$ is the point data domain of the sonified image depending on time. If duration of the sonification procedure is *T* seconds, then the final sonified image is $\tilde{D}(T)$

**Figure 1:**
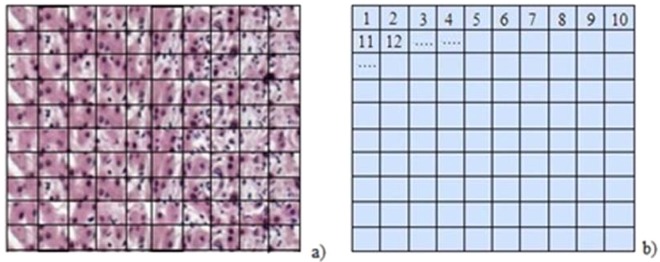
(**a**) A fictitious image with elements which may contain borders separating the colors and nuances, line and curves lines. (**b**) The matrix *D*.

This matrix *D* is shown in Fig. 1b. Each element of the matrix may contain color or nuances, interfaces or borders separating the colors and nuances, lines, curves and other objects (Fig. 1a).

Equation (7) is solved for sharp interface continuity conditions for both displacements and stresses. The reflections by the edges of the grids are removed by the Dirichlet and Neumann boundary conditions and a strongly attenuative buffer. The reflection coefficient is^22^

14 $${r}_{j}={(\frac{1-\cos {\rm{\theta }}}{1+\cos {\rm{\theta }}})}^{j},$$

where *j* is the degree of approximation, and *θ* is the incidence angle. For more than one component of displacement, the Dirichlet and Neumann conditions alternate components at the boundaries. When more than one boundary is nonreflecting, more solutions are added to eliminate multiple reflections.

After sonification, the point data domain of the sonified image $\tilde{D}=\{{\tilde{d}}_{1},{\tilde{d}}_{2},\ldots ,{\tilde{d}}_{N}\},{\tilde{d}}_{i}\in {R}^{N}$ may contain small blurred areas with cavities and white dots, due to the inaccuracies of the original images. The convertor *C*_2_ is applied to $\tilde{D}$ in order to fill these bad zones with colors and objects by prolonging through continuity of the solutions (9) in the adjacent areas and points. The converter *C*_2_ is defined in the next Section.

## Methodology

The problem to be investigated and solved in this paper can be formulated in three steps as:

1. Given a digital image *B*, a converter *C*_1_ is applied to *B* to construct the data matrix *D* as *C*_1_(*B*) = *D*;
2. The sonification operator *S* is apply to *D* to obtain the sonified image as $S(D,t)=\tilde{D}(t)$. If duration of the sonification procedure is *T* seconds, then the sonified image is $\tilde{D}=\tilde{D}(T)$.
3. A converter *C*_2_ is applied to $\tilde{D}$ to obtain the final sonified image *B*_*fin*_ as ${C}_{2}(\tilde{D})={B}_{fin}$.

Explanation of each path follows:

Path 1. The picture *B* is uniform sampled in a grid with equal size boxes *B* = {*b*_1_, *b*_2_, …, *b*_*N*_}, *b*_*i*_ ∈ *R*^*N*^. The sampling must be sufficiently fine with special care devoted to eliminate (or at least to keep under control) the sources of numerical errors.

The converter *C*_1_ is applied to *B* to obtain the point data *D* = {*d*_1_, *d*_2_, …, *d*_*N*_}, *d*_*i*_ ∈ *R*^*N*^ (Fig. 1b) as

15 $${C}_{1}:B\to D,\,\,{C}_{1}({b}_{j})=M(F,{b}_{j})={d}_{j},\,\,j=1,2,3,\ldots ,N$$

where *M*(*F*) is an alphabet map containing four filters *F*_*j*_, *i* = 1, 2, 3, 4, i.e. *F*_1_ the color and nuances filter, *F*_2_ the interfaces and borders separating the colors and nuances filter, *F*_3_ the line and curved lines filter, and *F*_4_ other objects that appears in the image filter (Fig. 1a). The filters are controlled by a code *J*_*fil*_ of minimizing the resolution loss and improving of the noise performance.

**Figure 2:**
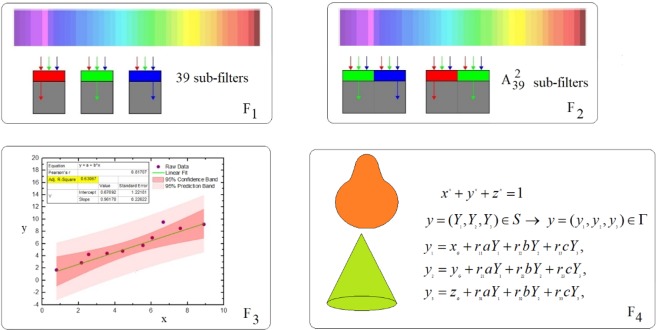
Scheme of the alphabet *M*(*F*).

The filters have ability to notice fine image details be it color or line, with no connection to pixel count or pixel density. Scheme of the alphabet *M*(*F*) is presented in Fig. 2.

Each filter contains a number of sub-filters *sF*_*ik*_, *i* = 1, 2, 3, 4, *k* = 1, 2, 3 …, *K*.

Filter *F*_1_ contains 39 sub-filters *sF*_1*k*_, *k* = 1, 2, 3 …, 39, for 39 color nuances. Each sub-filter captures a single color. Each color has a code *α*_*k*_, *k* = 1, 2, 3 …, 39.

Filter *F*_2_ contains ${A}_{39}^{2}$ sub-filters *sF*_2*j*_, *j* = 1, 2, …, ${A}_{39}^{2}$. Each interface has a code *β*_*k*_, *k* = 1, 2, …, ${A}_{39}^{2}$, obtained by interpolating of two codes *α*_*k*_, *k* = 1, 2, 3 …, 39.

The filter *F*_3_ contains a routine for linear, polynomial or nonlinear curve fitting along with validation of fit tests. The line or curve is introduced by points and the routine determines the most suitable equation for it.

The filter *F*_4_ identifies other objects from *B*. The shape Γ of the object is defined as the image of the unit *n*-sphere *S* of equation

16 $${x}^{n}+{y}^{n}+{z}^{n}=1,$$

through the affine transformation

17 $$y=({Y}_{1},{Y}_{2},{Y}_{3})\in S\to y=({y}_{1},{y}_{2},{y}_{3})\in \Gamma ,$$

18 $$\begin{array}{c}{y}_{1}={x}_{G}+{r}_{11}a{Y}_{1}+{r}_{12}b{Y}_{2}+{r}_{13}c{Y}_{3},\\ {y}_{2}={y}_{G}+{r}_{21}a{Y}_{1}+{r}_{22}b{Y}_{2}+{r}_{23}c{Y}_{3},\\ {y}_{3}={z}_{G}+{r}_{31}a{Y}_{1}+{r}_{32}b{Y}_{2}+{r}_{33}c{Y}_{3},\end{array}$$

where *r*_*ij*_ = *r*_*ij*_(ξ, ψ, ζ) are the components of rotation which transforms the coordinate axes into the principal axes of the sphere.

An inverse problem is applied to find the set of parameters (shape parameters) that define Γ, i.e. arbitrary center coordinates *x*_*G*_, *y*_*G*_, *z*_*G*_, principal axes *a*, *b*, *c*, the principal directions defined by Euler angles ξ, ψ, ζ and the exponent *n*. The advantage of this model is the small number of parameters needed to represent a shape.

Each filter works across a different algorithm.

2. The sonification operator *S*(*D*, *t*) defined by (13) is applied to *D* to obtain the sonified image $\tilde{D}$ as

19 $$S(D,t)=\tilde{D}(t)={F}_{s}(t)+\frac{{F}_{s}(t)}{1+{F}_{s}(t)},$$

where *F*_*s*_(*t*) is the generation sound force determined from the condition of minimum acoustic power *W*. By setting $\frac{\partial W}{\partial {F}_{s}}=0$, the function *F*_*s*_(*t*) is determined as

20 $${F}_{s}(t)=\mathop{\sum }\limits_{l=1}^{2}\frac{1}{2}[\frac{2{\rm{\pi }}}{{K}_{l}\sqrt{{m}_{l}}}{\mathop{\sum }\limits_{k=0}^{p}[\frac{{q}_{l}^{k+1/2}}{1+{q}_{l}^{2k+1}}{\rm{cn}}({m}_{l},\frac{{{\rm{\pi }}{\rm{\omega }}}_{l}t(k+1)}{2{K}_{l}})]}^{2}],$$

with

21 $$\begin{array}{c}{q}_{1}=\exp \,(-\,{\rm{\pi }}\frac{{K}_{2}}{{K}_{1}}),\,\,{q}_{2}=\exp \,(-\,{\rm{\pi }}\frac{{K}_{1}}{{K}_{2}}),\\ {K}_{1}={K}_{1}({m}_{1})+{\int }_{0}^{{\rm{\pi }}/2}\frac{du}{\sqrt{1-{m}_{1}\,{\sin }^{2}u}},\,\,{K}_{2}({m}_{2})={K}_{1}({m}_{1}),\,\,{m}_{1}+{m}_{2}=1.\end{array}$$

In (16) $\tilde{D}=\{{\tilde{d}}_{1},{\tilde{d}}_{2},\ldots ,{\tilde{d}}_{N}\},{\tilde{d}}_{i}\in {R}^{N}$ is the point data domain of the sonified image, and *t* is the sonification time.

3. After sonification, $\tilde{D}$ may contain small blurred areas with cavities and white dots due to the inaccuracies of the original images. We term these areas as damaged zones.

The convertor *C*_2_ has the role to fill the damaged zones with color and geometric lines, through continuity of solutions (9) of adjacent areas and neighboring points.

The converter *C*_2_ is applied to $\tilde{D}(T)=\tilde{D}$ to obtain the final sonified image *B*_*fin*_ = {*b*_1*fin*_, *b*_2*fin*_, …, *b*_*Nfin*_}, *b*_*ifin*_ ∈ *R*^*N*^, as ${C}_{2}(\tilde{D})={B}_{fin}$

22 $${C}_{2}:\tilde{D}\to {B}_{fin},\,{C}_{2}({\tilde{d}}_{j})=J({\tilde{d}}_{j})={b}_{jfin},\,j=1,2,3,\ldots ,N,$$

where

23 $$J({\tilde{d}}_{j})=\mathop{{\rm{\min }}}\limits_{{\rm{\delta }}P}||{\rm{\delta }}P-{\rm{\delta }}X|{|}^{2},$$

measures the distance between the solution in a damaged point and the solutions in its neighboring points. In (23), δ*X* is the solution in the damaged point, and δ*P* is the prolonged solution through continuity of the solutions in neighboring points. $J({\tilde{d}}_{j})$ determines the best solution in the damaged areas or points. Less spatial and color artifacts and better noise performance is assured by *C*_2_ compared with the existing schemes.

The filters are controlled by a code *J*_*fil*_ of minimizing the resolution loss and improving of the noise performance

24 $${J}_{fil}={{\rm{\min }}}_{{F}_{j},j=1,2,3,4}({{\rm{\alpha }}}_{c}{{\rm{\varepsilon }}}_{c}+{{\rm{\alpha }}}_{l}{{\rm{\varepsilon }}}_{l}+{{\rm{\alpha }}}_{I}{{\rm{\varepsilon }}}_{I}),$$

where ε_*c*_, *ε*_*l*_, ε_*I*_ measure the relative errors for identification of colors, the lines and curves, and the moment of inertia of objects, defined as

25 $$\begin{array}{c}{{\rm{\varepsilon }}}_{c}=\mathop{\sum }\limits_{j=1}^{N}(\frac{{C}_{1}({b}_{j})}{{d}_{j}}-1),\,{{\rm{\varepsilon }}}_{l}=\mathop{\sum }\limits_{j=1}^{N}(\frac{{C}_{2}({\tilde{d}}_{j})}{{b}_{jfin}}-1),\\ {{\rm{\varepsilon }}}_{I}={(\frac{{\sum }_{1\le i,j\le 3}{({I}_{ij}(S)-{I}_{ij}(\Gamma ))}^{2}}{{\sum }_{1\le i,j\le 3}{I}_{ij}^{2}(\Gamma )})}^{1/2},\,{I}_{ij}(S)=\frac{1}{5}{\int }_{S}{y}_{i}{y}_{j}{y}_{k}{n}_{k}dS.\end{array}$$

In (24), α_*c*_, α_*l*_, α_*I*_ are the associated weights.

## Applications

**Figure 3:**
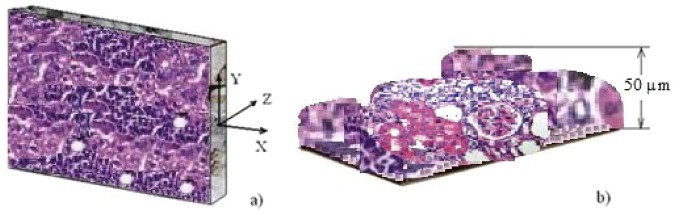
(**a**) A fictive rat liver sample. (**b**) The constituent’s size.

A sample of a fictive rat liver which exhibit changes in profile by severe loss of architecture and disturbances zones (between 10 and 50 μm) at the microscopic scale^23^, is shown in Fig. 3a. The size of constituents is displayed in Fig. 3b.

**Figure 4:**
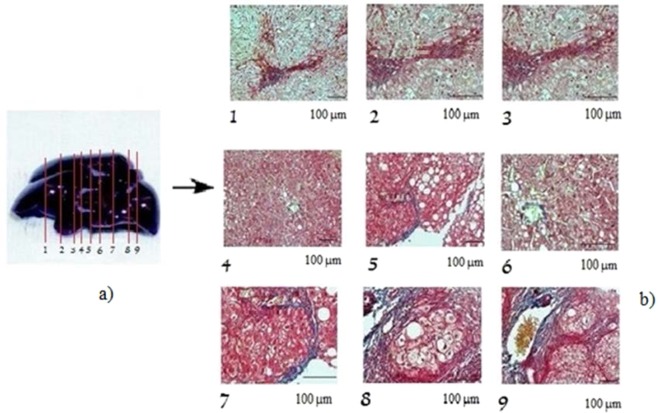
Nine sectional slices of a rat liver sample. (**b**) Digital images of cross-sectional slices.

The sonification operator (13) is exercised on fictive images of fibrotic rat liver samples inspired from an investigation of the effects of an extract of ginkgo biloba leaf against hepatic toxicity induced by methotrexate in rat^23,24^. The cross-sections of the rat liver are shown in Fig. 4.

For the first two applications, the simulations were performed for digital images of 1.600 × 1.200 pixels (length and width). Sonification time is 6 sec., *n* = 3 for the unit *n*-sphere *S*, and the weights in the code *J*_*fil*_ (α_*c*_, α_*l*_, α_*I*_) = (0.4, 0.4, 0.2).

The properties for the rat liver are: density *ρ*_0_ = 1.05 g/cm^3^, dissipation coefficients *b* = (*b*_1_, *b*_2_, *b*_3_) = (0.2, 0.2, 0.2) kg sec/m^2^, nonlinear coefficients β = (β_1_, β_2_, β_3_) = (0.3, 0.3, 0.3) sec.

**Figure 5:**
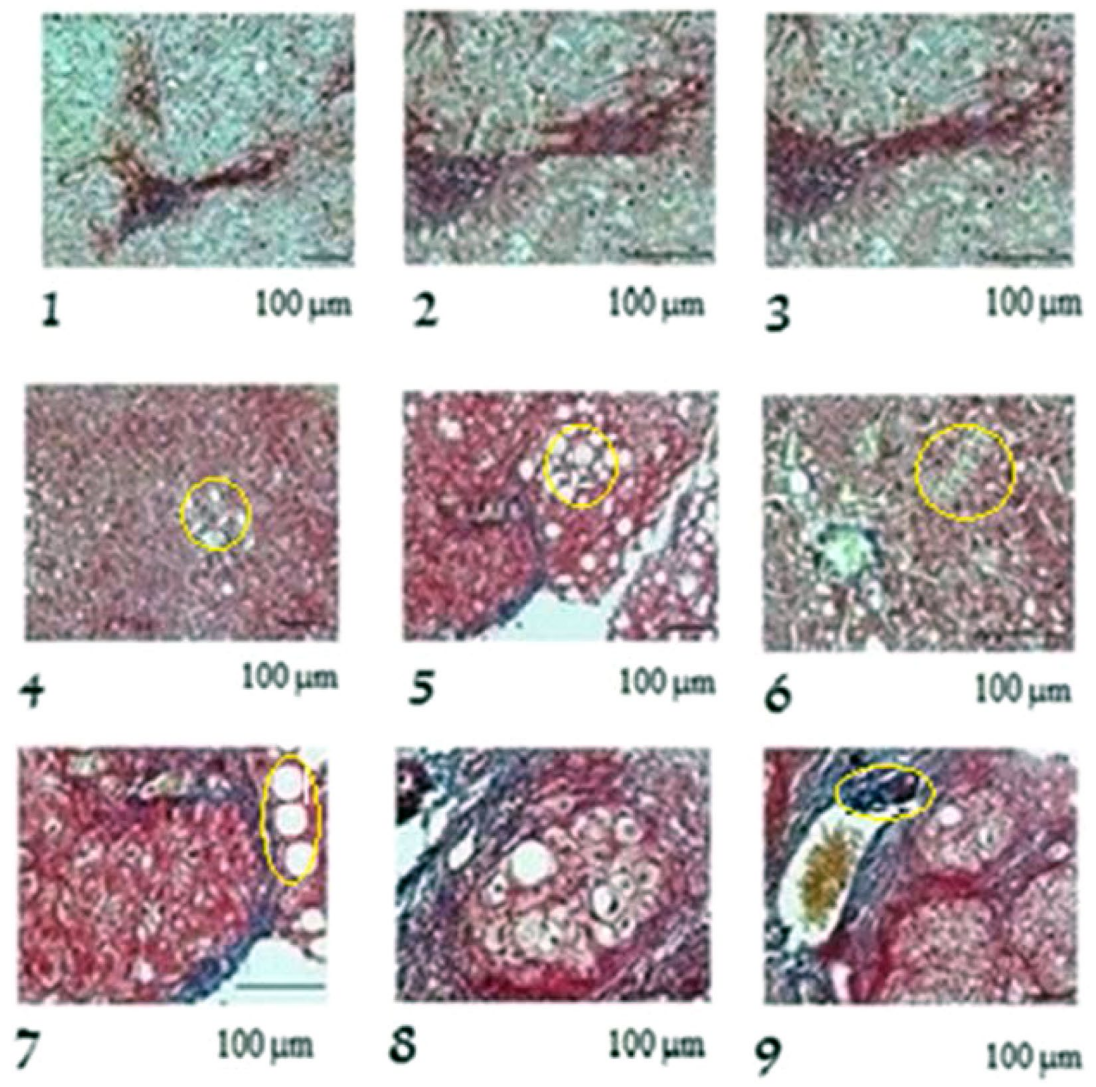
Cross-sectional slices of the sample after sonification. Yellow circles indicates the differences with the original images.

Figure 5 visualizes the new images obtained after sonification. By comparing these images to the original ones, some differences are highlighted in yellow in the last six images. Although there appears a tendency for the replication of images already known, our results show relevant details absent in the original images.

**Figure 6:**
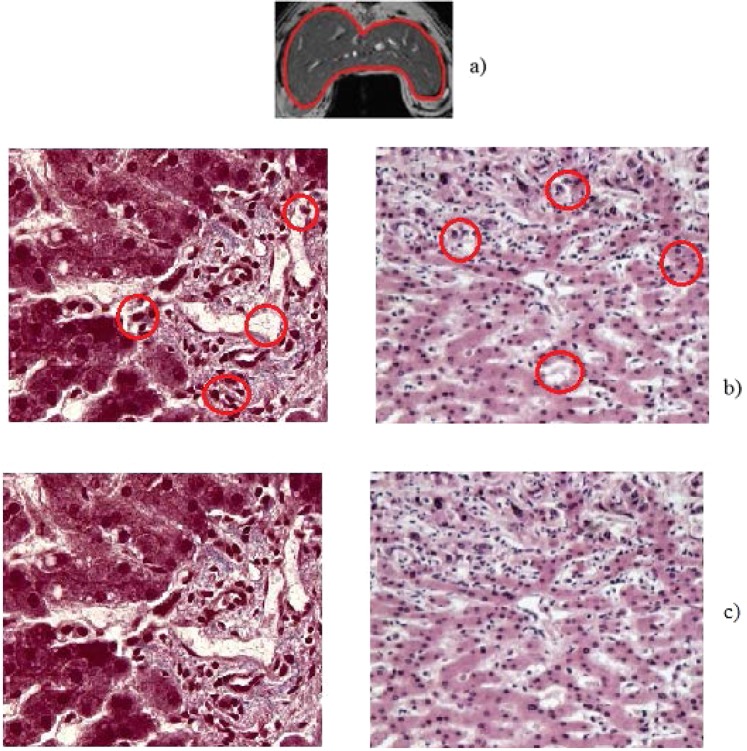
(**a**) The MR image of a liver rat. (**b**) Initially hidden details - red circles. (**c**) The hidden details were recovered by the sonification technique.

For the next exercise, we consider the work of Salameh^25^ which studies the detection of nonalcoholic steatohepatitis in the fatty rat livers by magnetic resonance (MR). This study is useful in the early detection of fibrosis in the at livers^26–28^. Figure 6a shows the MR image of a liver rat with strong hepatocellular damages. Some details are purposefully hidden (red circles in Fig. 6b). We see that the inverse sonification operator recovered all initially hidden details (Fig. 6c).

**Figure 7:**
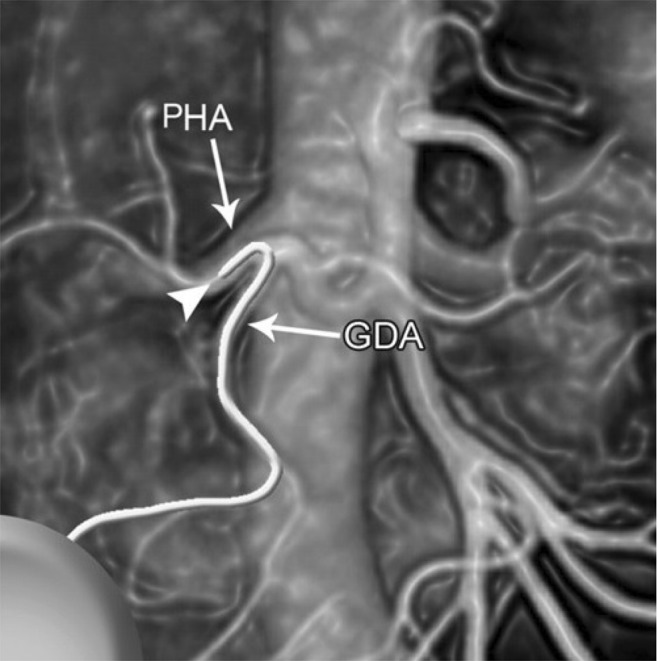
A possible location of the hepatic arterial infusion catheter^30^.

Another application is related to the hepatic arterial chemotherapy^29–32^. The knowledge of hepatic and biliary vascular maps is absolutely necessary for planning the surgical operation. A catheter must be inserted inside the gastroduodenal artery (GDA) to distribute the chemotherapy. A possible location of the hepatic arterial infusion catheter was discussed in^30^ and shown in Fig. 7.

**Figure 8:**
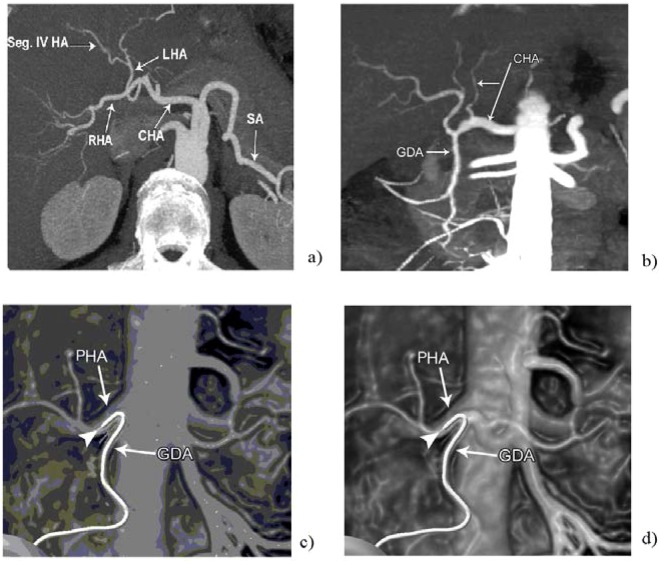
(**a**) CT image of the hepatic artery (CHA - common hepatic artery, LHA- left hepatic artery, RHA - right hepatic artery, SA - splenic artery, Seg IV HA - segment IV hepatic artery). (**b**) CT image of the left hepatic artery; (**c**) the image before sonification; (**d**) final sonified image.

Figure 8a shows the CT image of the hepatic artery (CHA- common hepatic artery, LHA - left hepatic artery, RHA - right hepatic artery, SA - splenic artery, Seg IV HA - segment IV hepatic artery)^30^. Figure 8b shows the CT image of the left hepatic artery^30^.

Our intension is to obtain an image by sonification of the image shown in Fig. 8c.

For last applications, the simulations were performed for digital images of 1.600 × 1.200 pixels (length and width). Sonification time is 7 sec., *n* = 3 for the unit *n*-sphere *S*, and the weights in the code *J*_*fil*_ (*α*_*c*_, *α*_*l*_, *α*_*I*_) = (0.4, 0.4, 0.2).

The properties for the rat liver are: density ρ_0_ = 1.12 g/cm^3^, dissipation coefficients *b* = (*b*_1_, *b*_2_, *b*_3_) = (0.2, 0.2, 0.2) kg sec/m^2^, nonlinear coefficients β = (β_1_, β_2_, β_3_) = (0.3, 0.3, 0.3) sec.

Final sonified image is shown in Fig. 8d. We see that it is identically to the one shown in Fig. 7.

**Figure 9:**
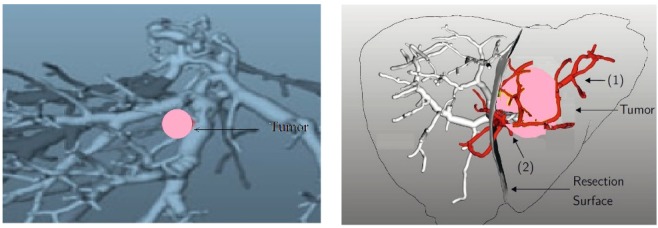
(**a**) The tumor location. (**b**) Vascular territory (1) and the vessel branches in the vicinity of the tumor (2).

The last application considers the case of a tumor (pink color) located near the portal tree of the vascular territory (Fig. 9a)^33,34^. The vascular territory (1) and the vessel branches in the vicinity of tumor (2) are shown in Fig. 9b.

**Figure 10:**
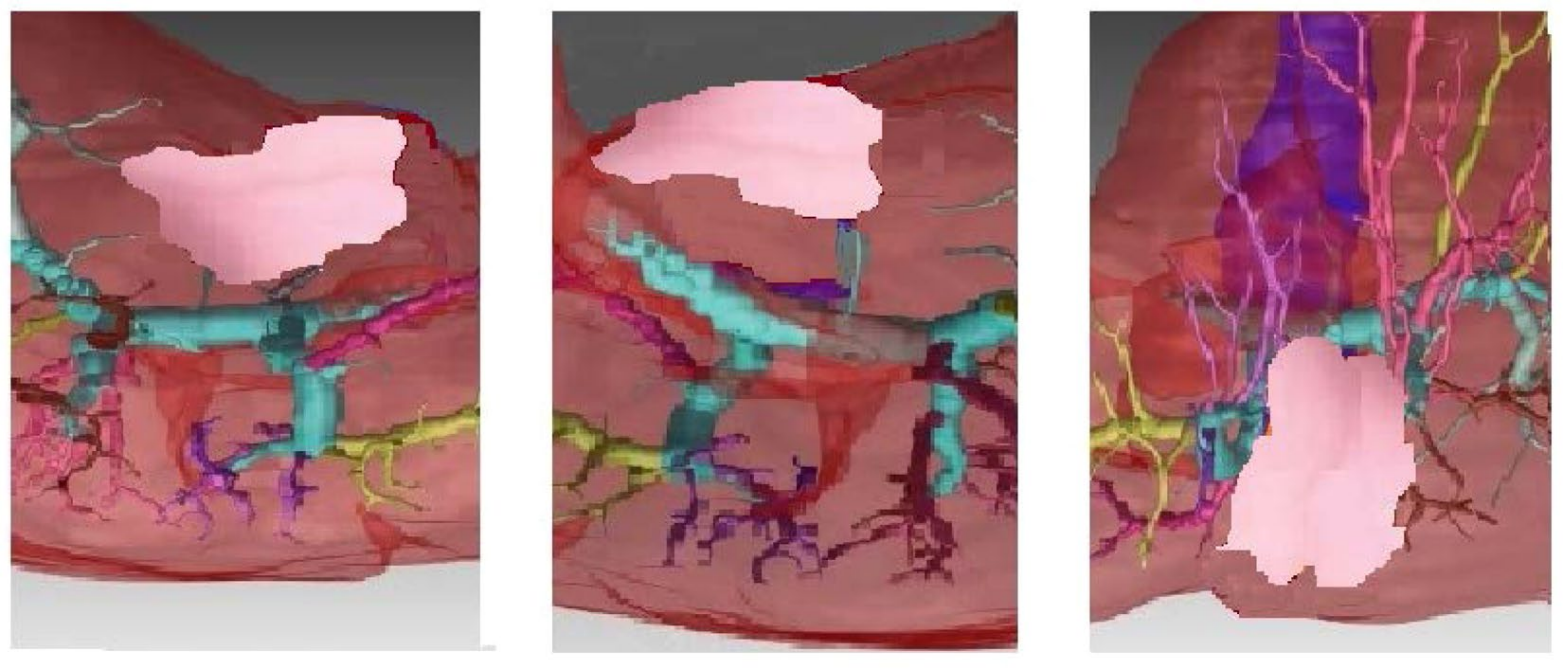
Sonified images in the vicinity of the tumor after sonification.

After sonification three images are obtained in Fig. 10 for the frontal, caudal and cranial views. New details on the tumor and surrounding areas are obtained and aditionally, the shape and size of the tumor is better visualized.

## Conclusion

The sonification operator proposed in this paper is converting the digital data field into sounds by using the nonlinear Burgers sound equation which is solved by cnoidal method. By inverting the sound into image, the result highlights hidden details in the image seen by the sound and not seen by the eyes. To show the efficiency of the sonification procedure and to verify the correctness of the results, we intentionally hide some details in the images before the sonification. The sonification operator has a positive effect in improving the accuracy in the medical imaging by capturing and detecting hard-to-see details in original images.

The advantages of the sonification refer to: (1) there is no distinction between the data time and the sonification time; (2) reflects indirectly the relationship between the propagation of sound through tissues and the structure of these tissues; (3) discovers new hard-to-find details in original medical images; (4) demonstrates the usefulness of image-sound and sound-image conversions in exploiting the medical imaging in diagnosis and surgery.
